# A Programmable Gain Calibration Method to Mitigate Skin Tone Bias in PPG Sensors

**DOI:** 10.3390/bios15070423

**Published:** 2025-07-02

**Authors:** Connor MacIsaac, Macros Nguyen, Alexander Uy, Tianmin Kong, Ava Hedayatipour

**Affiliations:** Department of Electrical Engineering, California State University Long Beach, Long Beach, CA 90803, USA

**Keywords:** PPG, wearable sensor, heart rate sensor, SpO_2_, wearable biosensors

## Abstract

Photoplethysmography (PPG) is a widely adopted optical technique for cardiovascular monitoring, but its accuracy is often compromised by skin pigmentation, which attenuates the signal in individuals with darker skin tones. This research addresses the challenge of skin pigmentation by developing a PPG sensor system with a novel gain calibration strategy. We present a hardware prototype integrating a programmable gain amplifier (PGA), specifically the OPA3S328 operational amplifier, controlled by a microcontroller. The system performs a one-time gain adjustment at initialization based on the user’s skin tone, which is quantified using RGB image analysis. This “set-and-hold” approach normalizes the signal amplitude across various skin tones while effectively preserving the native morphology of the PPG waveform, which is essential for advanced cardiovascular diagnostics. Experimental validation with over 70 human volunteers demonstrated the PGA’s ability to apply calibrated gain levels, derived from a first-degree polynomial relationship between skin pigmentation and red light absorption. This approach significantly improved signal consistency across different skin tones. The findings highlight the efficacy of pre-measurement gain correction for achieving reliable PPG sensing in diverse populations and lay the groundwork for future optimization of PPG sensor designs to improve reliability in wearable health monitoring devices.

## 1. Introduction

**Figure 1:**
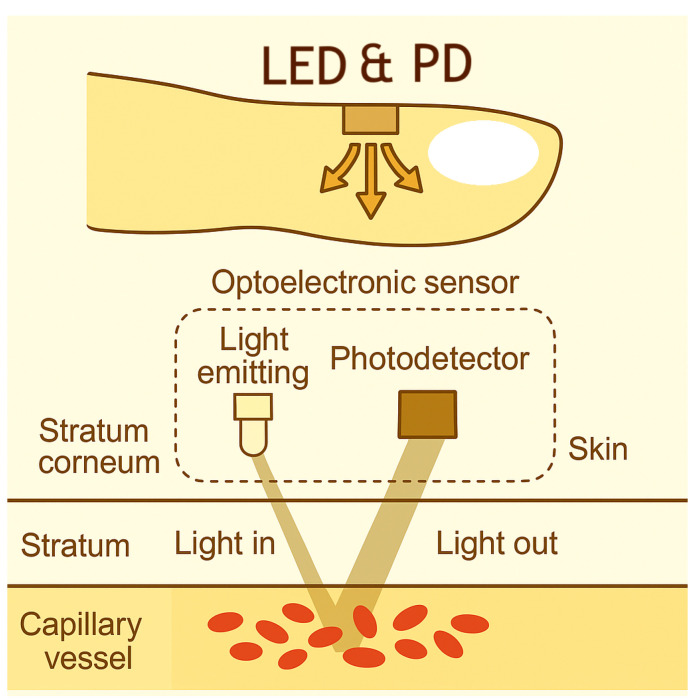
Schematic diagram of a reflective PPG sensor measurement setup. Light is emitted by an LED, penetrates the skin tissue, and the reflected light from blood vessels is captured by a photodetector (PD) [3].

Photoplethysmography (PPG) has become a prominent non-invasive optical technique for monitoring cardiovascular parameters, having been widely researched and developed over the past few decades. PPG sensors collect data by emitting light through the skin and interacting with blood vessels. The back-reflected light or transmitted light is then read and processed as a PPG signal, producing a waveform that can derive physical data such as heart rate and oxygen saturation (SpO_2_) [1]. Due to their non-invasive nature and ease of integration, PPG sensors are widely used in commercial products such as smartwatches (Apple Watches, Fitbits, etc.) and pulse oximeters. The light absorption characteristics of hemoglobin, which vary depending on the wavelength of light and its oxygenation state, are utilized in this process (as illustrated in Figure 1). The Beer–Lambert law is fundamental to this process, relating light attenuation to the concentration of chromophores like oxygenated and deoxygenated hemoglobin, which is crucial for SpO_2_ calculation [2].

**Figure 2:**
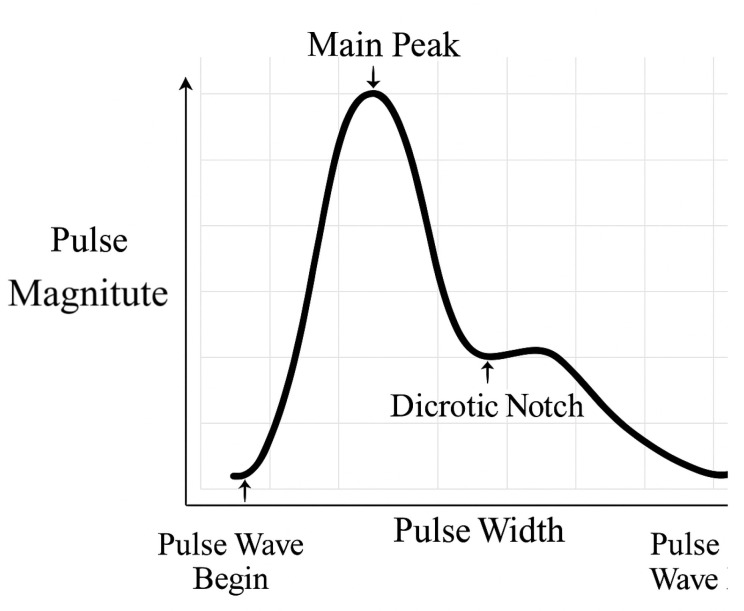
Key features of a typical PPG waveform, including the systolic peak (main peak), dicrotic notch, pulse width, and pulse amplitude. These features provide insights into cardiovascular status [4].

**Figure 3:**
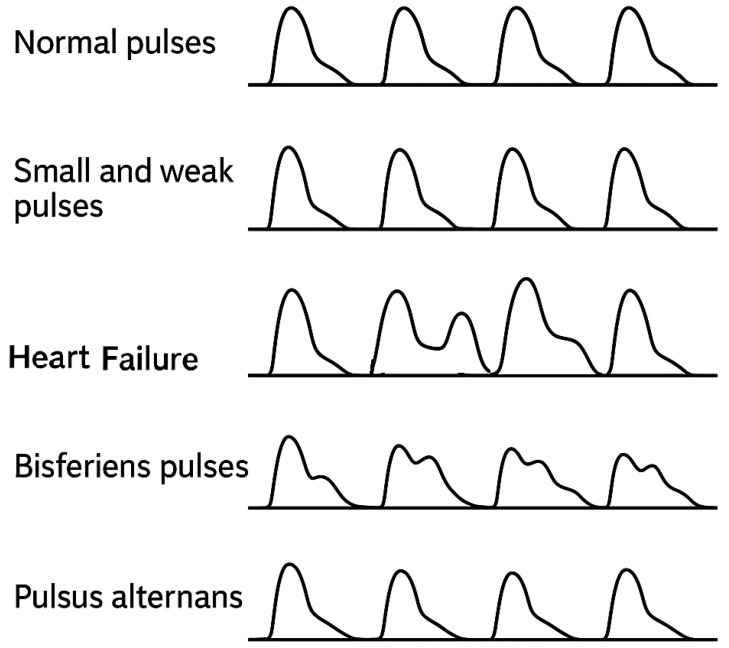
Illustrative examples of different PPG pulse morphologies and their potential association with various cardiovascular conditions. For instance, a “small and weak” pulse can indicate different physiological states compared with a “normal” pulse [5,6].

A typical PPG waveform has four key features (Figure 2): the main peak, dicrotic notch, pulse width, and pulse amplitude. In a healthy cardiovascular system, the main peak typically measures about twice the amplitude of the dicrotic notch. Deviations from this ratio may indicate underlying health issues, thereby reinforcing the importance of accurate waveform interpretation. Figure 3 illustrates several PPG waveforms associated with different health conditions. A “normal pulse” shape generally indicates healthy cardiovascular function. Deviations, such as “small and weak pulses” where the main peak’s amplitude is significantly reduced relative to the dicrotic notch, may suggest conditions such as heart disease. “Large and bounding pulses” could result from exercise or pathology. Bisferiens pulses, with dual systolic peaks, may indicate serious cardiac issues, and pulsus alternans—alternating amplitudes—are linked to life-threatening complications.

More recently, SpO_2_ has gained public attention due to its pivotal role in assessing respiratory health during the COVID-19 pandemic. Reduced respiration from COVID-19 infection lowers SpO_2_ levels to potentially fatal thresholds. Medical guidelines suggest that SpO_2_ levels below 92% warrant medical attention, with levels below 88% considered critical [7,8,9]. Given its clinical relevance, accurate and timely detection of SpO_2_ is vital, particularly for diseases that compromise respiratory function. PPG sensors, which deliver SpO_2_ and heart rate measurements to the user, must therefore operate with high fidelity. Even slight inaccuracies in SpO_2_ readings can have significant clinical implications, contributing to the cautious adoption of some PPG-based devices for high-stakes medical decision-making.

A primary challenge to PPG sensor accuracy is the influence of various variables that many current designs do not adequately address, such as skin pigmentation, tattoos, movement, nail polish, and environmental lighting [10,11]. These factors influence the back-reflected light read by the system’s photodiode, which in turn affects the PPG signal. Both the pulsatile (AC) component, which reflects pulsatile changes due to cardiac cycles, and the baseline (DC) component, which represents baseline blood volume in the tissue, are susceptible to these interferences [2]. Consequently, variations in these components can ultimately skew measurements that are essential for determining heart rate and hemoglobin levels [12]. In other words, these PPG systems must be designed to preserve these waveform distinctions. However, if any of the environmental or physical variations mentioned cause a healthy waveform to resemble a pathological one, without compensation, the sensor’s diagnostic value could be compromised.

Numerous studies have identified factors that limit the accuracy of PPG sensors. Wearable devices, in particular, suffer from motion artifacts due to their placement on the wrist and fingers—mobile parts of the body exposed to both sedentary and dynamic activity. These artifacts distort the waveform, leading to amplitude spikes and corrupted readings [13,14]. Skin pigmentation and environmental lighting also introduce noise. Study [15] found that darker skin tones exacerbate errors in SpO_2_ measurements at low oxygen levels. At 60–70% saturation, participants with darker pigmentation experienced an average overestimation of 3.56 ± 2.45%, whereas those with lighter tones showed only a 0.37 ± 3.20% discrepancy. This variation underscores the urgency of addressing skin tone bias in sensor design. The study also highlighted that factors like tattoos and nail polish can further impact measurement reliability.

Similarly, research by Bazurto et al. [16] investigated the impact of pigmentation on PPG readings. Their study utilized artificial skin phantoms of varying tones to simulate different levels of pigmentation. The findings demonstrated a consistent pattern: lighter skin resulted in higher output voltage readings, while darker skin tones yielded lower output voltage readings. This inverse relationship between pigmentation level and signal amplitude was observed across different red light intensities.

The COVID-19 crisis also accelerated interest in remote health monitoring, particularly through telehealth platforms. In this context, video photoplethysmography (vPPG) has emerged as a promising technique, using video rather than physical contact sensors to derive vital signs. However, as demonstrated in “Effect of Ambient Lighting and Skin Tone on Estimation of Heart Rate and Pulse Transit Time from Video Plethysmography” [17], both skin pigmentation and lighting conditions significantly affect the accuracy of both vPPG and traditional PPG systems. While improved lighting conditions can help mitigate some inaccuracies, these studies suggest that such measures may not fully eliminate skin tone-related discrepancies in vPPG measurements.

Together, these studies highlight the pervasive impact of skin pigmentation on the performance of both contact and remote PPG systems. The implications extend beyond academia. For instance, wearable industry leaders like Apple and Garmin have faced criticism for sensor inaccuracies related to tattoos and darker skin tones. In response, these companies have introduced software-based correction algorithms, increased LED intensity, and improved sampling rates [18]. Support resources provided by Garmin [19] and Apple [20] also aim to educate users on pigmentation-related discrepancies.

Other studies have also focused on improvements in heart rate detection algorithms. Ahn et al. [21] proposed integrating a Viterbi algorithm with a hidden Markov model, boosting measurement accuracy to approximately 98%. This methodology assigns reliability scores to light intensity samples gathered by PPG sensors and filters out unreliable data. The proposed system operates with negligible latency (0.2 ms per sample) and a modest increase in power consumption (roughly 1 mA), making it a practical solution for real-time monitoring.

Advancements have also been made in integrating PPG technology into commercial-grade wearables. Lee et al. [22] introduced organic thin-film transistor (OTFT)-based amplifiers for dual-function measurements: arterial pulse detection and sweat pH analysis. Although promising, this design yielded a heart rate error margin of ±1 beat per minute when compared with standard electrocardiogram (ECG) machines. For medical-grade applications, even this level of deviation may be deemed excessive and warrants further refinement.

This research aims to address the challenge of skin pigmentation-induced variability in PPG signal amplitude. Our primary objective is to develop and validate a system that normalizes PPG signals to achieve consistent output characteristics across users with diverse skin tones by performing a one-time gain calibration. Normalization, in this context, involves adjusting the sensor’s output to counteract the attenuating effects of melanin, thereby ensuring that the measured signal amplitude is less dependent on skin pigmentation.

To achieve this, we propose a system that performs a one-time gain calibration based on an individual’s skin tone at the beginning of a measurement. This “set-and-hold” approach was deliberately chosen over continuous real-time automatic gain control (AGC), which can distort the PPG waveform’s diagnostic features by altering its morphology. Our method preserves the crucial characteristics of the waveform, including its amplitude, width, and key morphological features, ensuring the clinical utility of the signal remains intact.

The system was implemented through a complete data acquisition pipeline using a programmable gain amplifier (PGA). The PGA’s gain is set based on the participant’s skin tone, determined from RGB image analysis, ensuring that amplification is tailored to compensate for melanin-specific light absorption. By compensating for signal attenuation without compromising intrinsic waveform morphology, our approach seeks to enhance the accuracy and reliability of PPG sensing, paving the way for more equitable and consistent use of these sensors in diverse populations and clinical applications.

This paper is organized as follows: Section 2 outlines the methodology used to acquire PPG signals from human participants, including the design of the testing environment and data acquisition protocols. Section 3 describes the steps taken to clean, segment, and analyze the raw signals, ensuring reliable input for further analysis. In Section 4, the use of a PGA to normalize signal amplitude across varying skin tones is detailed, along with the algorithmic logic used to maintain waveform integrity. Section 5 presents the outcomes of the experiments, including performance comparisons before and after gain normalization. Section 6 interprets the results, evaluates the effectiveness of the approach, and addresses limitations and future improvements. Finally, Section 7 summarizes the key contributions of the study and highlights its potential impact on the development of more equitable and accurate PPG-based health monitoring systems.

## 2. Data Collection

The primary objective of this phase was to collect accurate and representative PPG signals from a cohort of 70 human participants to investigate the effects of skin pigmentation and to test our gain compensation strategy. The data collection procedure was structured to ensure consistency, reliability, and control over environmental and physiological variables that could influence signal.

### 2.1. PPG Data Collection

**Figure 4:**
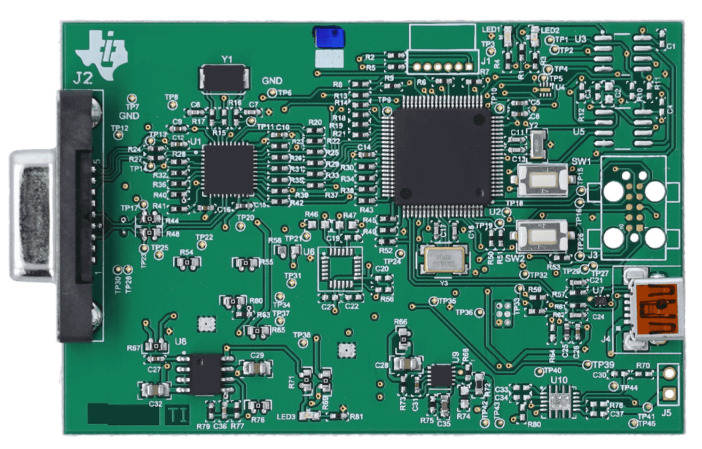
TI AFE 4400 commercially available board to use as a pulse oximeter sensor [23].

**Figure 5:**
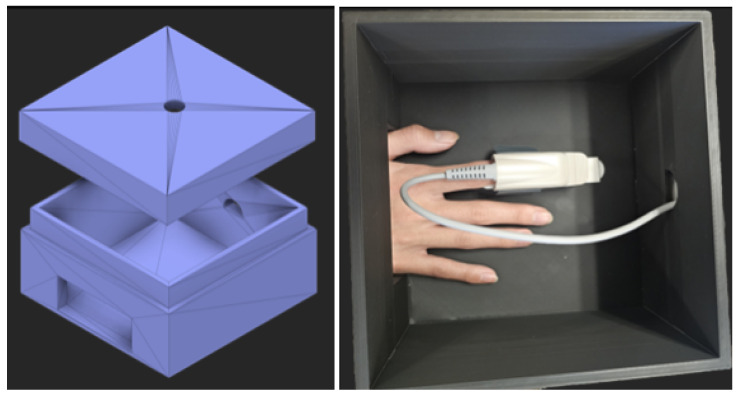
Experimental setup showing the 3D-printed enclosure designed to minimize ambient light and motion artifacts. (**Left**) Exterior view of the enclosure. (**Right**) A participant’s hand is inserted into the enclosure, with their finger in the PPG clip sensor connected to the TI AFE4400 EVM.

High-fidelity PPG data were acquired using a Texas Instruments AFE4400 evaluation module (EVM) (Figure 4), a specialized analog front-end for optical biosensing applications, manufactured in Dallas, TX, USA [23]. The AFE4400 was configured with a standard finger-clip PPG sensor. To minimize external interference, the sensor was positioned in a fixed location within a custom-designed, 3D-printed enclosure (Figure 5). The enclosure was designed in SolidWorks, 2023 educational version and was 3D printed using a Bambu Lab 3D printer, based in Austin, TX, USA, serving to shield the sensor from ambient light and reduce motion artifacts by providing a stable platform for the participant’s hand. The enclosure also featured dedicated wiring ports, an opening for participants to insert their hands, and an opening for a webcam used for skin pigmentation assessment.

Participants were instructed to sit comfortably and remain still, with their figure placed in the PPG sensor clip inside the enclosure. During the resting phase, the TI AFE 4400 sensor captured the PPG waveform recorded at baseline, ensuring initial recordings reflected resting heart activity. Data acquisition was managed through the accompanying TI software, which also ensured standardized recording parameters and consistent file management. Following the baseline measurement, each participant engaged in brief physical activity to elevate heart rate (e.g., jumping jacks, walking up and down stairs), allowing the system to capture PPG waveforms under increased physiological demand. Throughout all trials, consistent lighting conditions within the laboratory and uniform sensor placement and data recording protocols were maintained to facilitate a focused analysis of the impact of skin pigmentation on PPG signal characteristics.

### 2.2. Skin Pigmentation Measurement

To ensure reproducible quantitative assessment of skin pigmentation, a standard 1080p webcam interfaced to a Raspberry Pi 4 was used under a highly controlled protocol. Each participant’s hand was photographed within the testing enclosure, which shielded the hand from variable ambient lighting and provided a consistent illumination environment. The webcam was maintained at a fixed distance and angle, and its settings (e.g., white balance, exposure) were held constant across all sessions to prevent automatic adjustments. The captured images were then processed using a k-means clustering algorithm implemented in Python (v3.13).

**Figure 6:**
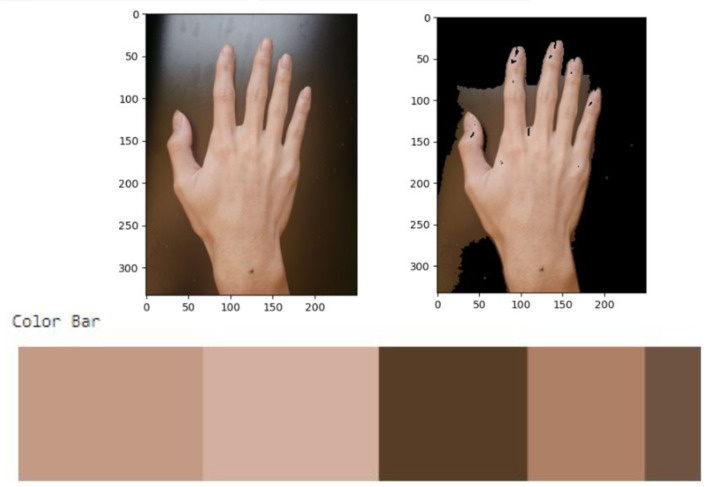
Illustration of the skin pigmentation extraction process. An image of the participant’s hand is processed using a k-means clustering algorithm to identify the dominant RGB color value, which serves as a quantitative measure of skin tone.

This algorithm first segmented the hand region from the image background. Then, k-means clustering was applied to the RGB pixel values within the hand region to identify dominant color clusters, making the measurement robust against minor local variations such as freckles or veins. The RGB value of the most dominant cluster was extracted and recorded as a quantitative representation of the participant’s skin pigmentation (an example of this process is shown in Figure 6). This objective measure of skin tone was then used for correlating with PPG signal characteristics and for calibrating the gain system.

### 2.3. Participant Demographics and Ethical Considerations

To contextualize the PPG and pigmentation data, each participant completed a digital survey prior to testing. The survey collected information on heart conditions, gender, age, fitness level, and other relevant demographic and health-related factors. These metadata were collected to provide context for the physiological measurements and to aid in the interpretation of data variability.

The study protocol was approved by the Institutional Review Board (IRB) of California State University, Long Beach (Protocol ID: 2126009-2). All participants provided informed consent before engaging in the study. To ensure participant safety and data integrity, all equipment, including the testing enclosure and PPG sensor, was sanitized between sessions. All information is recorded anonymously, and all images are removed after the RGB value is recorded into our database.

## 3. Data Processing

Following data acquisition, the raw PPG signals and associated survey data underwent a series of processing steps to prepare them for analysis and use in the dynamic gain system.

### 3.1. PPG Signal Pre-Processing and Feature Extraction

The raw PPG signal contains both a large, slowly varying DC component, related to baseline tissue and non-pulsatile blood absorption, and a smaller, pulsatile AC component, which is modulated by cardiac-induced blood volume changes. The AC component, which carries the essential physiological information, was isolated for analysis. The TI AFE4400’s analog front-end includes an ambient light cancellation circuit and filtering stages that effectively remove the DC offset to extract the pulsatile signal.

**Figure 7:**
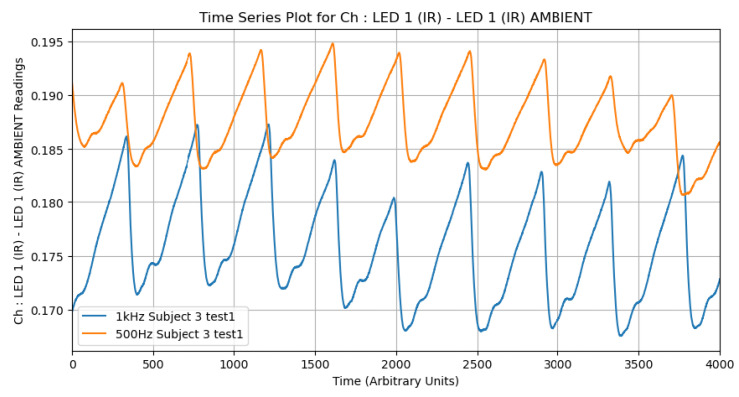
Example of PPG signal segments from Subject 3 processed with different low-pass filter settings (e.g., 500 Hz and 1 kHz). The choice of filter parameters can impact the amplitude and morphology of the resulting waveform.

Raw PPG signals obtained from the TI AFE4400 often contain noise from various sources, including high-frequency electronic noise and baseline wander. To enhance the signal-to-noise ratio, appropriate filtering techniques are essential. As an initial investigation, we compared the effects of different low-pass filter settings (e.g., 500 Hz vs. 1 kHz cutoff frequencies) on the PPG waveform characteristics using data from a subset of subjects. It was consistently observed that the 500 Hz filter produced higher amplitude signals. This behavior suggests that lower-frequency filtering better preserves the pulsatile components of the waveform while attenuating higher-frequency noise, making it particularly valuable for detecting subtle physiological variations (Figure 7). For the main analysis, specific filtering parameters from the TI AFE4400 EVM v1.4 software’s default configuration for SpO_2_ were used, which typically include bandpass filtering to isolate the relevant frequency range of the PPG signal (e.g., 0.5 Hz to 5 Hz for heart rate).

The data collected for the PPG was processed using custom Python scripts, leveraging libraries such as Pandas and Scipy for data manipulation. Key steps included:

- **Signal segmentation:** Isolating relevant portions of the PPG waveform corresponding to baseline and elevated heart rate periods.
- **Peak detection:** A peak detection algorithm was employed to identify systolic peaks in the AC component of the PPG signal. Each detected peak corresponds to a heartbeat.
- **Heart rate calculation:** Heart rate (HR) in beats per minute (BPM) was calculated from the intervals between successive systolic peaks.
- **Amplitude extraction:** The peak-to-peak amplitude of the AC component ($V_{pp}$) and the mean of the DC component ($V_{mean}$) were extracted for both red and infrared PPG signals. These values are crucial for SpO_2_ calculation and for analyzing signal strength.

The processed physiological data, including calculated HR, $V_{pp}$, $V_{mean}$, and derived SpO_2_ values, were stored along with subject identifiers for subsequent merging with survey and skin pigmentation data. The data visualization was performed using Matplotlib.

The initial analysis of this processed data reaffirmed the existing literature in that skin pigmentation significantly affects PPG signal amplitude, with darker pigmentation generally leading to lower signal strength and potentially less accurate readings if not compensated for. This highlighted the necessity for the dynamic gain adjustment system developed in this research.

### 3.2. Survey Database Processing

**Figure 8:**
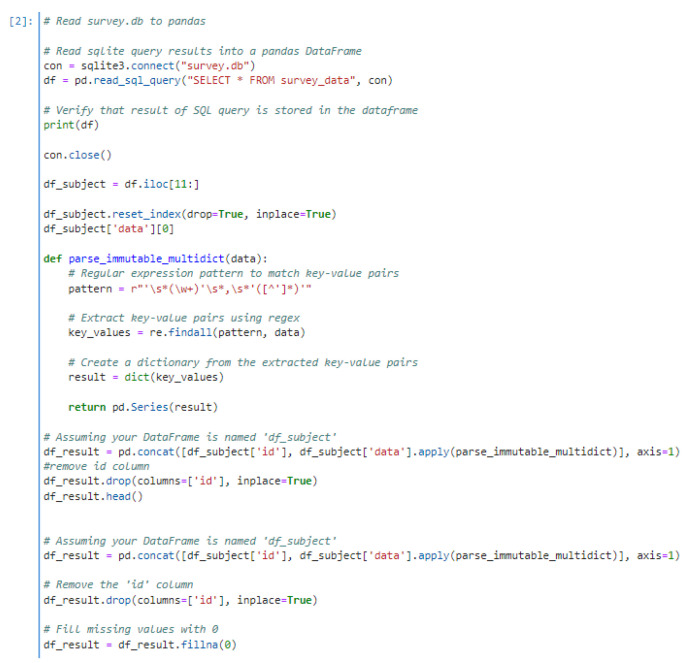
Python code snippet illustrating the process of reading the SQLite survey database (.db file) into a Pandas DataFrame for further manipulation and merging with physiological data.

Two primary datasets were used in this study: a structured survey database and physiological measurements recorded using the TI AFE 4400 evaluation board. The survey data were stored in a .db SQLite database file, which was programmatically read into a Pandas DataFrame using Python (Figure 8).

**Figure 9:**
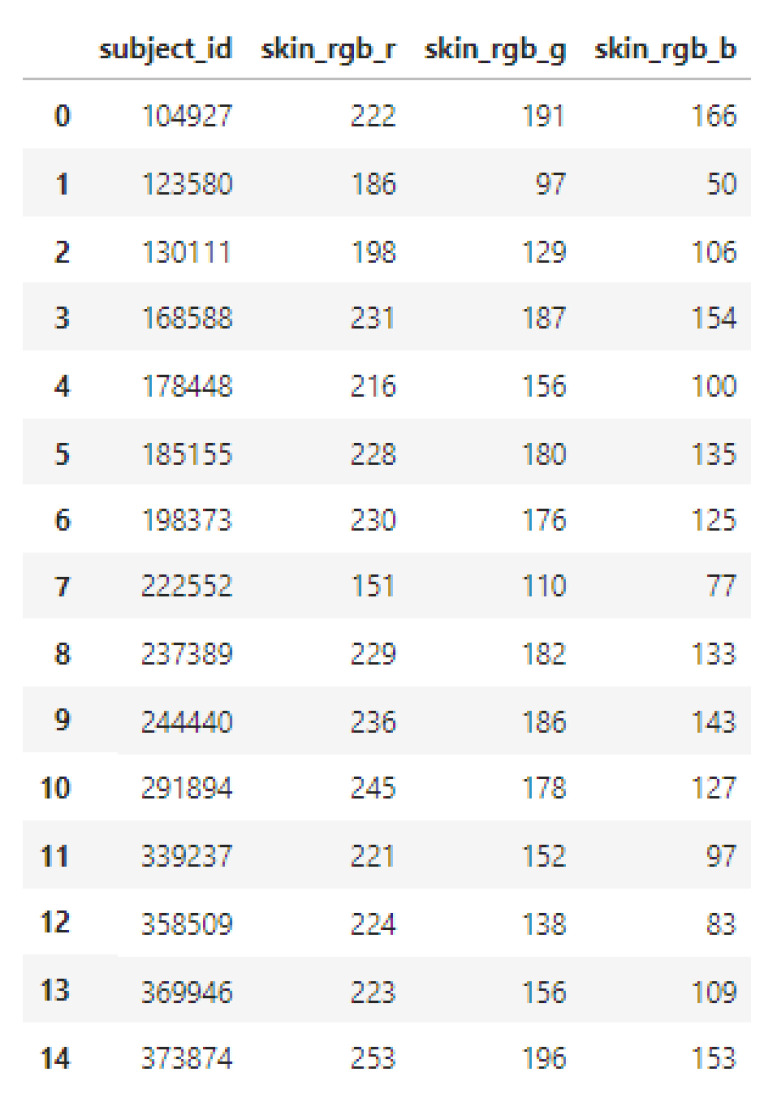
Representation of the filtered Pandas DataFrame containing subject IDs and their corresponding mean RGB skin pigmentation values, extracted from the survey database and image processing pipeline.

The full survey DataFrame contained various fields including subject ID, gender, age, and skin pigmentation values (RGB). For analysis purposes, only the RGB skin pigmentation values and subject IDs were retained; all other metadata were dropped to streamline the dataset (Figure 9).

### 3.3. TI Physiological Data Processing

**Figure 10:**
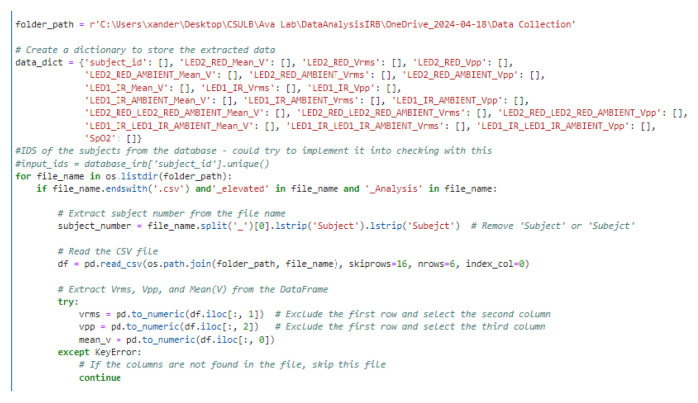
Python code snippet demonstrating the directory traversal and file filtering logic used to locate and parse relevant CSV data files generated by the TI AFE4400 evaluation software.

The second dataset consisted of physiological signals recorded using the TI AFE 4400 evaluation board. These data were organized into CSV files stored in a directory structure, with each file labeled with a subject ID and an indication of physical activity level (“elevated” or “base”). Python scripts recursively traversed the folders to locate files of interest, filtering based on naming patterns (see Figure 10).

Each CSV file was parsed by skipping the first sixteen lines (headers) and reading six lines of signal data. Key metrics such as peak-to-peak voltage (Vpp), root-mean-square voltage (Vrms), and mean voltage (Vmean) were extracted and stored in a structured DataFrame. A subject ID field was added based on the filename for alignment with the survey data.

SpO_2_ was computed based on the principle that oxygenated hemoglobin (HbO_2_) and deoxygenated hemoglobin (Hb) absorb red and infrared light differently [24]. The “ratio of ratios” (*R*), which relates the pulsatile (AC) and non-pulsatile (DC) components of the PPG signal at both wavelengths, is calculated as:

(1) $$R=\frac{{\mathrm{AC}}_{\mathrm{Red}}/{\mathrm{DC}}_{\mathrm{Red}}}{{\mathrm{AC}}_{\mathrm{IR}}/{\mathrm{DC}}_{\mathrm{IR}}}$$

where ${\mathrm{AC}}_{\mathrm{Red}}$ and ${\mathrm{DC}}_{\mathrm{Red}}$ are the AC and DC components of the red light signal, and ${\mathrm{AC}}_{\mathrm{IR}}$ and ${\mathrm{DC}}_{\mathrm{IR}}$ are the corresponding components for the infrared signal.

SpO_2_ is typically estimated from *R* using a linear empirical formula of the form ${\mathrm{SpO}}_{2}=A-B\cdot R$, where the coefficients A and B are determined by calibration against a reference co-oximeter. While the TI AFE4400 documentation suggests initial coefficients of A=110 and B=25, we observed that this produced physiologically implausible results (e.g., >100%) with our specific sensor setup.

Therefore, for the purpose of this study, we made an empirical adjustment to constrain the SpO_2_ estimates to a more realistic range, resulting in the following formula:

(2) $${\mathrm{SpO}}_{2}\left(\%\right)=100-5\cdot R$$

It is critical to acknowledge that this is a simplified, non-calibrated formula. Its purpose in this work is not for clinical diagnosis but rather to establish a functional relationship between the optical signal and a physiological parameter for testing our gain calibration methodology. For clinical-grade accuracy, a robust calibration procedure would be essential.

**Figure 11:**
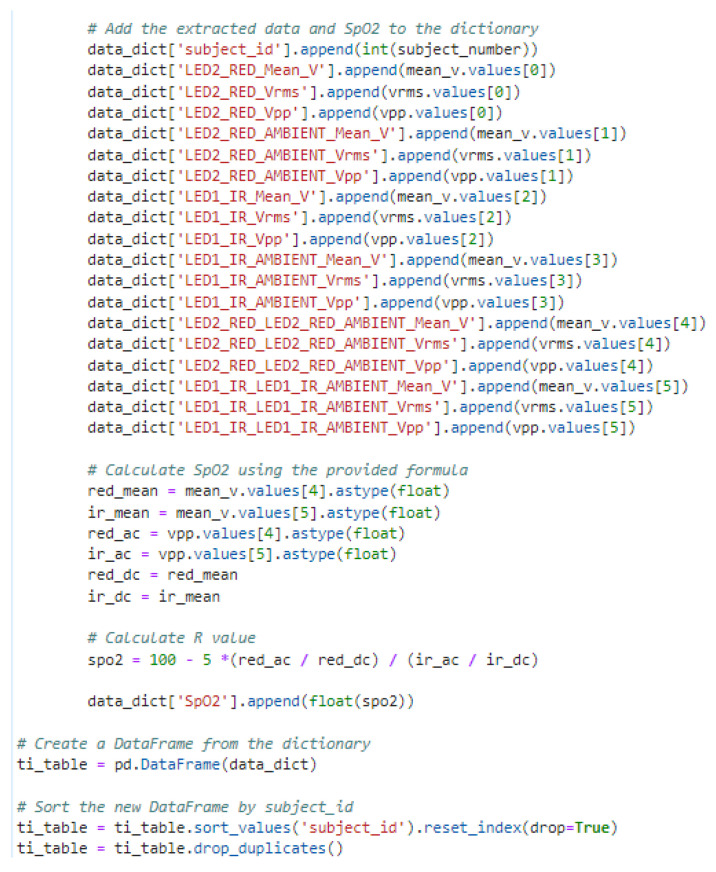
Python code snippet illustrating the assembly of processed TI AFE4400 signal data (e.g., AC/DC components), calculation of SpO_2_ using the empirical formula, and subsequent merging with the survey-derived skin pigmentation data.

The extracted metrics, including HR and calculated SpO_2_, were consolidated into a master DataFrame and merged with the filtered survey data and skin pigmentation data using the subject ID as the common key (an example of this processing is shown in Figure 11).

## 4. Skin Tone-Based Gain Calibration System

To address the signal attenuation caused by varying skin pigmentation, we designed and implemented a system that performs a one-time gain calibration at the start of a measurement. This “set-and-hold” strategy adjusts the amplification of the PPG signal based on the participant’s skin tone and then maintains that fixed gain for the duration of the measurement. This approach is designed to normalize signal amplitude without distorting the waveform’s essential diagnostic features.

### 4.1. System Architecture and Workflow

**Figure 12:**
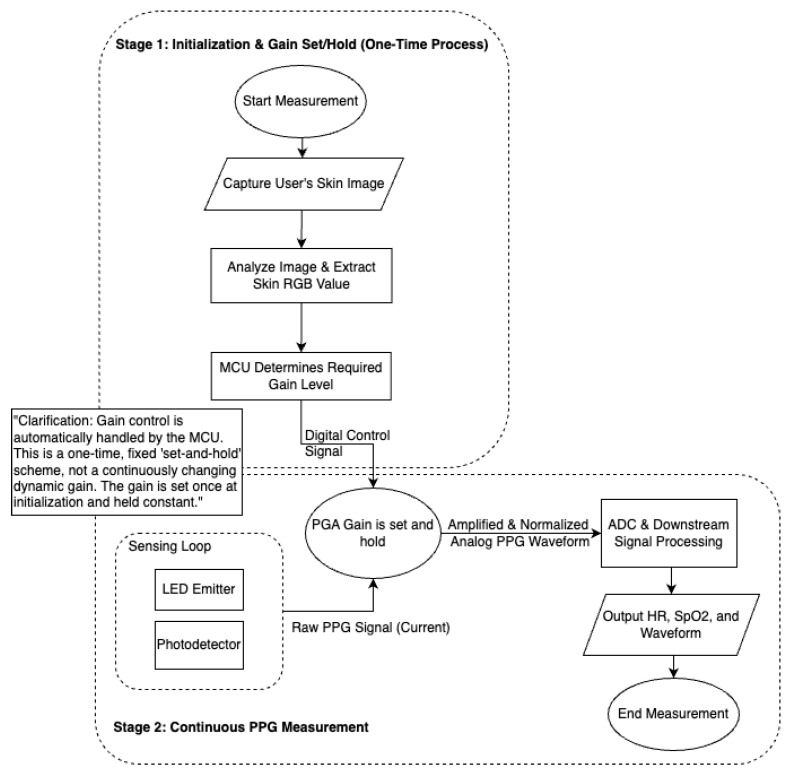
Overall system architecture for the PPG sensor with automated one-time gain calibration. The process is divided into an initial “set-and-hold” phase, where the gain is fixed based on skin tone, and a continuous measurement phase that uses the calibrated gain.

The overall architecture of our system is illustrated in Figure 12. The workflow is divided into two distinct stages: a one-time initialization and gain set/hold phase and a continuous PPG measurement phase. The entire process is automated by a microcontroller to ensure consistency and ease of use.

While the “set-and-hold” architecture is deliberately chosen for this study to prioritize waveform fidelity, future iterations of this work will explore a hybrid real-time gain control system. Such a system could use this initial calibration to set a baseline gain and then make minor, slow adjustments to account for transient factors like changes in pressure or perfusion, aiming to achieve the benefits of real-time adaptation without introducing the artifacts that corrupt diagnostic features.

The operational workflow, as depicted in the diagram, is realized by the core system components. The core of the hardware is the PGA circuit, built around the Texas Instruments OPA3S328 operational amplifier. This op-amp features integrated switches that allow for selectable feedback resistors, thereby changing the transimpedance gain. A Seeeduino Xiao microcontroller is used to control these switches.

The workflow steps are:

1. **Skin tone capture and analysis:** An image of the user’s skin is captured and analyzed to extract its dominant RGB value (see Section 2).
2. **Automated gain selection:** The Seeeduino Xiao microcontroller uses the skin’s red (R) channel value and a pre-defined calibration curve (see Section 5) to automatically determine the required gain level.
3. **Gain set and hold:** The microcontroller sends a digital control signal to the PGA, which sets the gain and holds it constant for the entire measurement session.
4. **Continuous measurement and output:** The raw PPG current from the sensor is continuously amplified by the PGA using the fixed gain, producing a normalized analog waveform ready for downstream processing.

**Figure 13:**
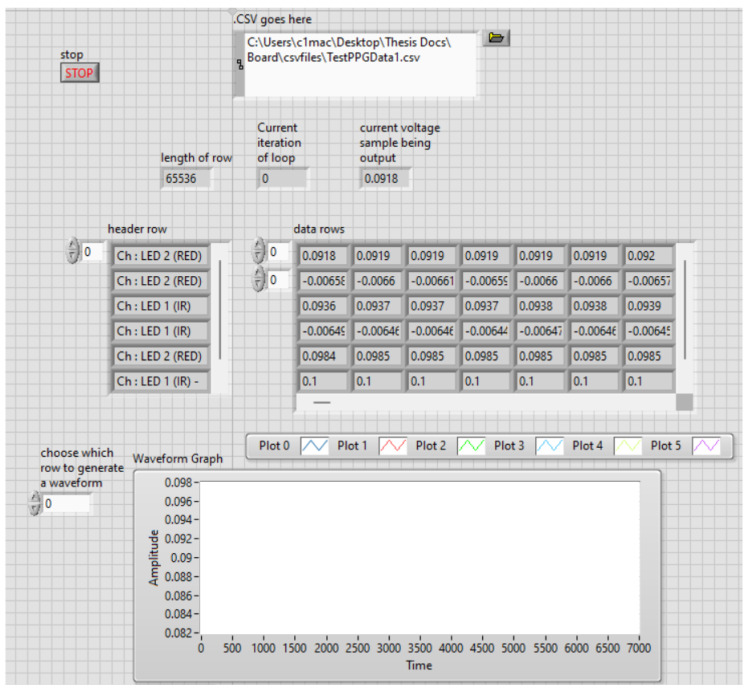
LabVIEW user interface for replaying stored PPG data as an analog waveform via the MyDAQ device. This allows for controlled input to the PGA system.

**Figure 14:**
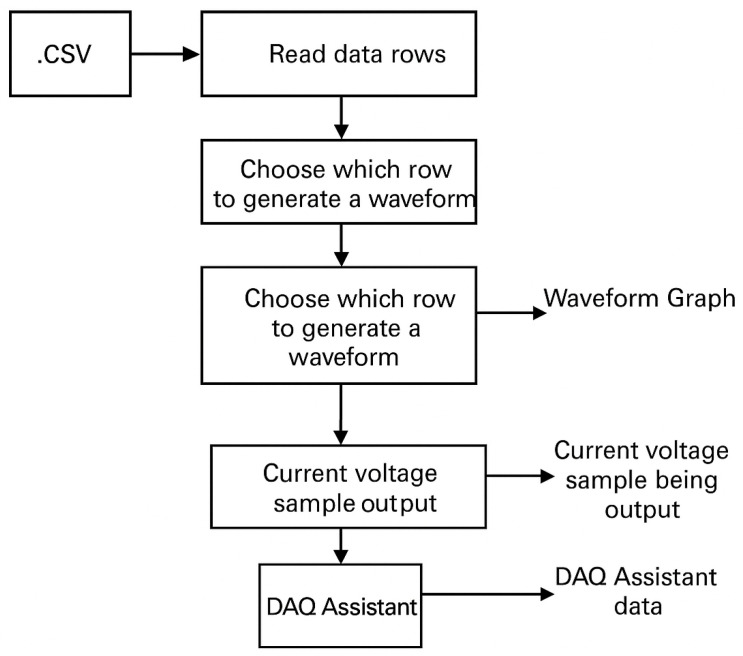
Block diagram of using LabVIEW user interface for replaying stored PPG data as an analog waveform via the MyDAQ device.

For the purposes of system validation, the input PPG signal was replayed using a National Instruments MyDAQ and LabVIEW, as shown in Figure 13 and Figure 14. This allowed us to test the PGA’s response with controlled, repeatable inputs.

### 4.2. PGA Circuit Design and Stability

**Figure 15:**
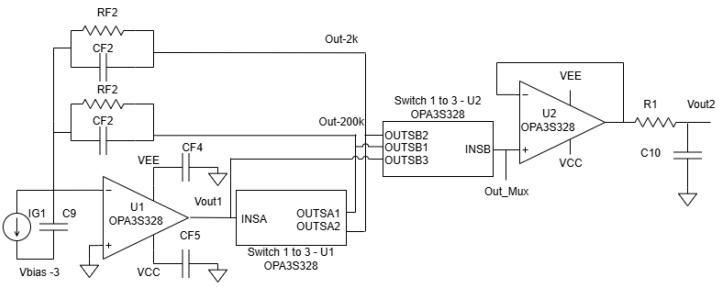
Detailed schematic of the PGA implementation using the OPA3S328 circuit. The circuit features two primary feedback paths (RF1/CF1 and RF2/CF2) selected by microcontroller signals (‘A1_CTRL’, ‘A2_CTRL’) to set the gain.

The programmable gain amplifier is the core of our calibration system. As shown in the detailed schematic in Figure 15, the design is based on the OPA3S328 integrated circuit, which includes a transimpedance amplifier (U1A) and a series of controllable switches. The gain is controlled by a Seeeduino microcontroller, which sends digital signals to the ‘A1_CTRL’ and ‘A2_CTRL’ pins to select one of two primary feedback paths. The specific component values used in this implementation are listed in Table 1.

Maintaining stability in a TIA circuit is crucial. The stability of the TIA circuit is ensured by managing the loop gain, which is defined as the product of the op-amp’s open-loop gain (AOL) and the feedback factor (β). Stability is achieved when AOL equals 1/β, a condition that varies with frequency. The critical frequency $F_{c}$, where these two curves intersect, must be carefully considered in the design.

Capacitor values $C_{f}$ must satisfy bandwidth constraints. For a desired closed-loop bandwidth of 500 kHz:

- For $R_{f1}=200\;k\Omega$, $C_{f1}<1.6$ pF.
- For $R_{f2}=2\;k\Omega$, $C_{f2}<159$ pF.

Parasitic capacitance from active switches and a series resistance (typically 90 Ω) must also be considered, as they can reduce the phase margin. At $C_{f1}=1.6$ pF, the phase margin is approximately 54.6°, whereas at $C_{f2}=159$ pF, it drops to 35.8°. To maintain a margin above 35°, a more conservative $C_{f2}$ value of 50 pF is used.

The op-amp’s rail-to-rail output (RRO) stage must also be protected. If the output approaches supply rails too closely, performance may degrade. A buffer margin of 200 mV is recommended by the manufacturer [25]. This constraint limits the allowable resistance in the feedback network. For worst-case diode current and switch resistance:

- For $I_{DMax}=20$ μA and $R_{ON}=125\;\Omega$, $R_{F}<34.8\;k\Omega$.
- For $I_{DMax}=2$ mA and $R_{ON}=125\;\Omega$, $R_{F}<2.28\;k\Omega$.

The selected resistor values fall within these constraints, ensuring stable amplifier operation without encroaching upon RRO limits. This dynamic gain design, integrated with real-time signal adaptation, provides a robust framework for improving the fidelity of PPG signals across diverse patient populations.

**Figure 16:**
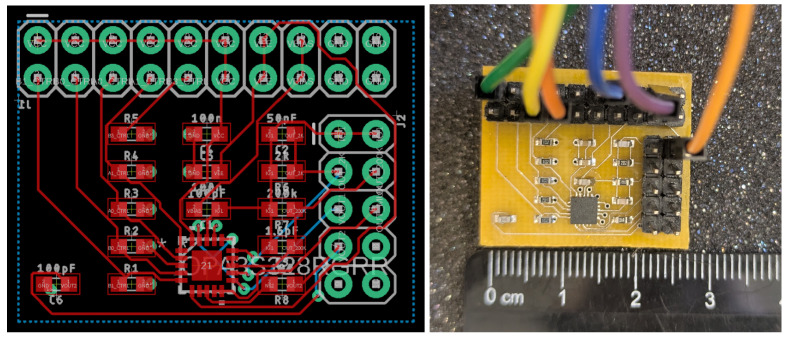
Photograph of the custom-designed PCB used for implementation and testing.

The overall system, including the PGA and microcontroller, was implemented on a custom-designed printed circuit board (PCB) shown in Figure 16. Initial validation of the switch control and gain stages was performed on this hard PCB prototype before integrating it with the MyDAQ-based signal replay system for testing with the collected human PPG data.

## 5. Experiment Validation and Results

The following testing procedures were conducted in order to confirm the ability to control the variability of the PGA into commercial PPG sensor designs to mitigate inaccuracies caused by various factors, particularly skin pigmentation. The analysis focused on assessing the performance of the PPG sensors with and without the PGA under different testing protocols.

### 5.1. Calibration of Gain Levels Based on Skin Pigmentation

**Figure 17:**
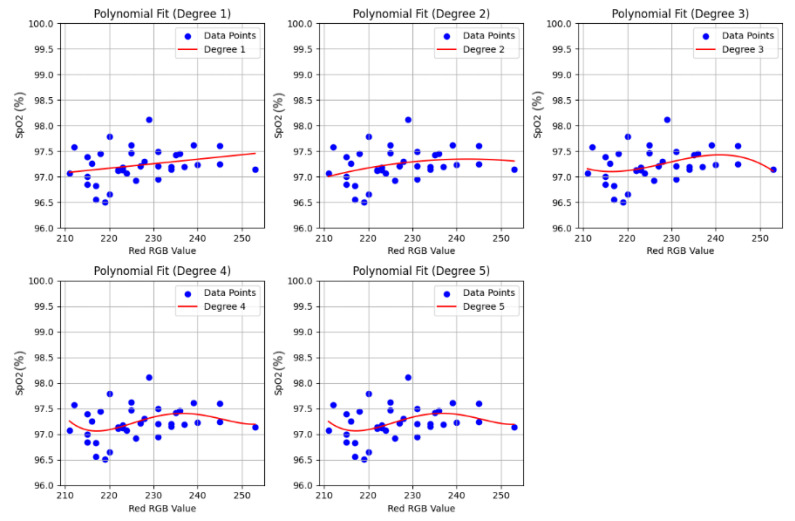
Dot plots showing the relationship between calculated SpO_2_ values and the red channel of skin RGB values from 70 participants. Various polynomial fits (Degrees 1 through 5) are overlaid. A first-degree polynomial was chosen for simplicity in defining gain thresholds.

The relationship between skin pigmentation (quantified by the red channel of RGB values from hand images) and the measured SpO_2_ values (calculated using Equation (2)) from the 70 participants was analyzed to establish a calibration basis for the dynamic gain system. As shown in Figure 17, which plots SpO_2_ against the red RGB value, there is considerable scatter. For the purpose of gain calibration, we focused on the general trend that darker skin often corresponded to signals that might require more amplification to achieve a consistent output level compared with lighter skin.

Based on this data-driven approach, the participants’ red RGB values were categorized into three tiers. Each tier was mapped to a specific gain setting in the PGA, as detailed in the gain selection logic in Table 2. These tiers and their corresponding hardware configurations were programmed into the Seeeduino microcontroller, ensuring that a higher gain (200 k V/A) was applied for darker skin tones and a lower gain (2 k V/A) for medium-toned skin. For the lightest skin tones where signal attenuation was minimal, the gain could be effectively bypassed.

While various polynomial fits were explored (Figure 17), a first-degree (linear) polynomial fit was selected for its simplicity. To define the gain tiers, we analyzed the distribution of the red channel RGB values from our 70-participant cohort and segmented the population into three similarly sized groups, or tertiles. Based on this data-driven approach, the participants’ red RGB values (ranging from approximately 210 to 252 in our cohort) were categorized into three tiers, each assigned a different gain factor by the PGA, as detailed in Table 2.

### 5.2. PGA Functionality Test: Switch Control and Gain Verification

Initial testing of the hard PCB prototype focused on verifying the correct operation of the PGA’s switching mechanism and its ability to deliver different gain levels.

A sinusoidal 1 kHz, 200 mVpp was used as an input, connected in series with a 5 kohm resistor to create a 4 μA current input. MyDAQ was then used as a waveform generator connected to a resistor in series, which served as the input current source. Using an external voltage source, 1 V was applied to control the ON and OFF status of each switch. The output was measured to demonstrate that the PGA could successfully manipulate the gain, providing a variable output voltage in response to the simulated input current.

**Figure 18:**
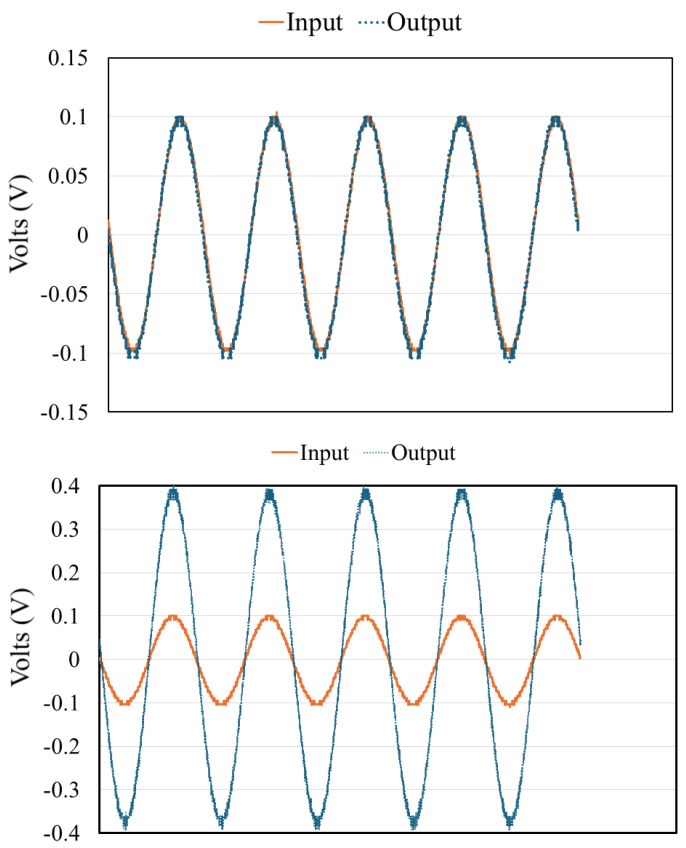
Oscilloscope capture showing an example of PGA gain verification. Yellow trace: input signal (scaled for representation). Blue trace: amplified output signal from the PGA. This example illustrates a gain of approximately 3.8×. (Note: replace with a clear, well-annotated image showing distinct input and output for a specific gain setting).

For example, to test a high-gain setting (intended for darker skin tones), the switches corresponding to a larger feedback resistor (e.g., $R_{F,high}=200\;k\Omega$) were activated. With a 4 μA input current, the expected output voltage would be $V_{out}=I_{in}\times R_{F}=4\;\mu A\times 200\;k\Omega =800\;\mathrm{mV}$. The measured output voltage was observed on an oscilloscope (Figure 18). Similarly, lower gain settings were tested by selecting smaller feedback resistors. These tests confirmed that the PGA could reliably switch between different gain levels as controlled by the microcontroller. For instance, a measured gain of approximately 3.8× was observed for one setting when a 4× gain was targeted, indicating reasonable performance close to the design value.

### 5.3. Effect of Calibrated Gain on PPG Signal Consistency Across Skin Tones

To evaluate the effectiveness of the one-time gain calibration in compensating for pigmentation-related signal attenuation, we compared the relationship between skin pigmentation and PPG signal amplitude under three conditions: base (resting), elevated (post-exercise), and dynamically adjusted gain.

**Figure 19:**
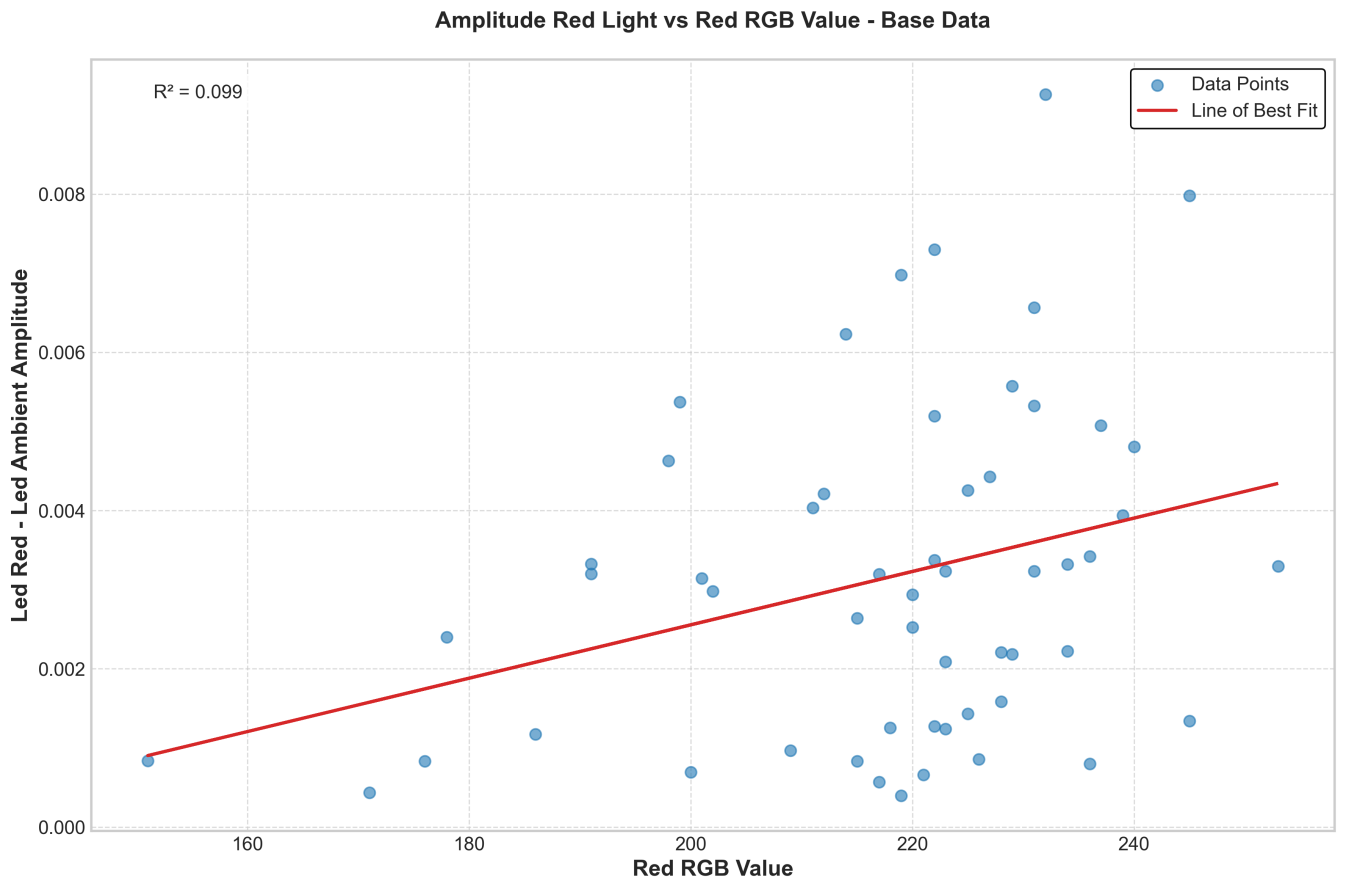
Amplitude of red light signal vs. red RGB value—base condition.

**Figure 20:**
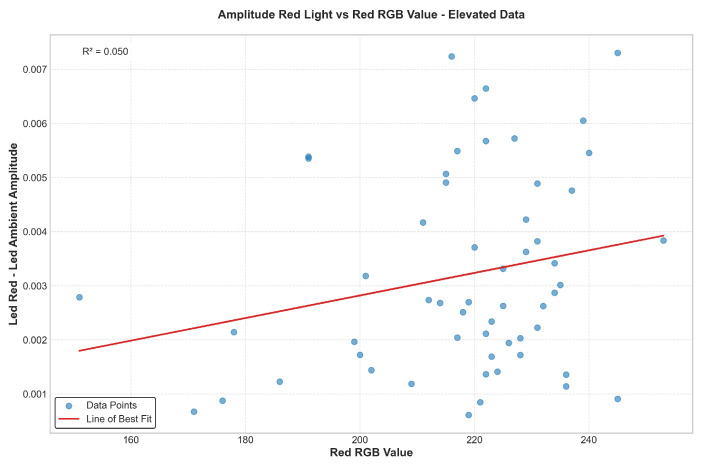
Amplitude of red light signal vs. red RGB value—elevated condition.

Figure 19 and Figure 20 illustrate a positive correlation between skin pigmentation—quantified via the red channel of the RGB value—and PPG signal amplitude. Specifically, individuals with lower red RGB values (indicating darker skin pigmentation) consistently exhibit lower measured signal amplitudes. This trend is evident in both elevated and base data, with the slope of the linear regression line indicating signal attenuation correlated with increased melanin absorption.

**Figure 21:**
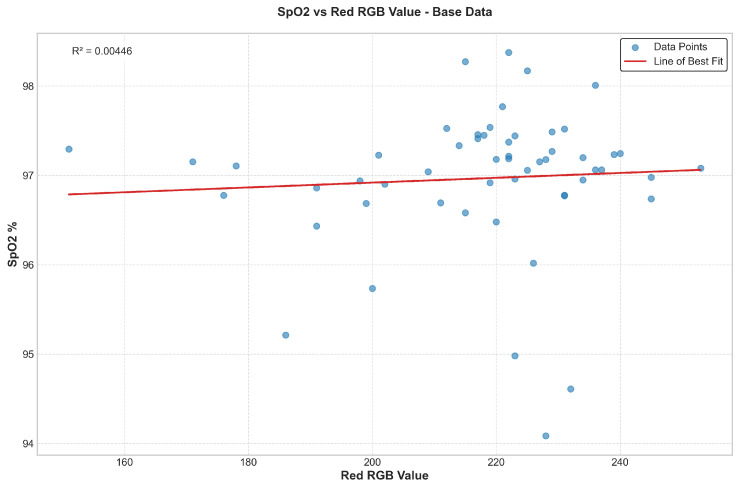
SpO_2_ vs. red RGB value after the calibrated gain implementation.

To mitigate this disparity, the “set-and-hold” gain calibration was implemented. As shown in Figure 21, applying the appropriate gain based on skin pigmentation significantly reduced the dependence of amplitude on pigmentation. This is demonstrated by a reduced slope in the linear regression and a lower R-squared value, indicating decreased variance explained by pigmentation alone. In effect, the calibrated gain approach flattens the amplitude response across pigmentation levels, standardizing the signal regardless of melanin content.

**Figure 22:**
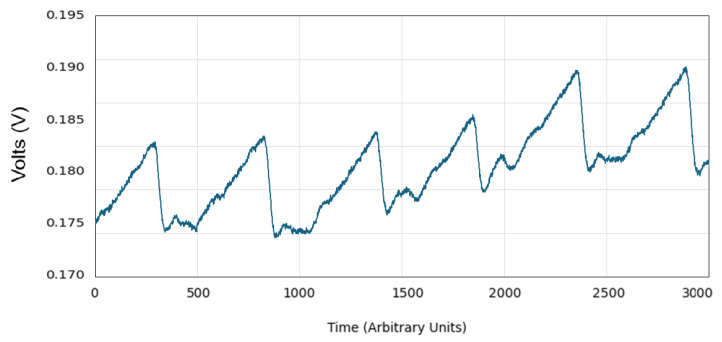
Heart rate signal after the calibrated gain implementation.

To evaluate the effectiveness of the gain compensation method signal amplitude, and SpO_2_ measurements were analyzed across participants with varying skin pigmentation levels. After applying the calibrated gain, signal amplitude across skin tones was equalized within ±0.0008 V, demonstrating significant improvement in consistency. The coefficient of variation (CV) across groups was reduced from 37.9% to 13.5%. The post-gain regression exhibits a 91% reduction in slope and near-zero R_2_ value, confirming that skin pigmentation had a negligible impact on signal amplitude after correction. Additionally, waveform the seen in Figure 22 and after gain adjustment are presented to illustrate that key morphological features—such as the systolic peak and dicrotic notch—are preserved, supporting the diagnostic integrity of the signal across varying pigmentation levels.

These results confirm that one-time gain calibration improves signal equity across skin tones. By reducing pigmentation-dependent attenuation, the system enhances the reliability of PPG measurements and enables more accurate physiological monitoring for a diverse population. Although the primary focus of this study was to address signal attenuation due to skin pigmentation, the gain adjustment principle would potentially be extended to mitigate other sources of signal degradation. Factors such as tattoos, which also increase light absorption, or low-perfusion areas could benefit from a similar gain calibration. However, this study did not experimentally isolate or test these other variables, and further research is required to validate the efficacy of the system under these conditions.

## 6. Discussion

The results of this study highlight the importance and feasibility of normalizing PPG signal amplitudes to mitigate the influence of skin pigmentation on sensor accuracy. Our data confirm previous findings that darker skin pigmentation lead to a to a reduced signal amplitude due to increased light absorption, which can significantly distort physiological readings such as heart rate and SpO_2_. By implementing a PGA with a one-time gain calibration controlled through skin tone data, we were able to significantly reduce signal variability across participants with differing skin tones while preserving the waveform morphology critical for diagnostic use.

The test results indicated a significant improvement in the consistency of the output voltage when the PGA was integrated into the PPG sensor design. The following observations were made:

- **Light skin pigmentation**: The output voltage remained stable, reflecting a consistent minimal deviation from the expected readings.
- **Medium skin pigmentation**: The PGA effectively adjusted the gain to compensate for the reduced back-reflected light, resulting in a consistent output voltage similar to that of light skin pigmentation.
- **Dark skin pigmentation**: The most notable improvement was observed in the readings of subjects with darker skin pigmentation. The PGA’s ability to boost the signal strength compensated for the lower back-reflected light intensity, producing output voltages comparable with those from lighter skin tones.

These findings collectively highlight the efficacy of the PGA-based normalization system in addressing the longstanding performance disparities observed in optical-based sensors across diverse populations. By implementing gain adjustments tailored to skin pigmentation, the system successfully bridges a critical equity gap in PPG signal acquisition.

A key advantage of our “set-and-hold” calibration, applied once at initialization, is the preservation of the PPG waveform’s morphological integrity. This was a deliberate design choice. Unlike continuous real-time gain adjustments, which can introduce artifacts by altering the relative amplitudes of the systolic peak and dicrotic notch, our method ensures the inherent shape of the signal is maintained. Such distortions from real-time systems can mask or even mimic pathological conditions, compromising the signal’s diagnostic value beyond simple heart rate extraction. By setting the gain only once, our system supports diagnostic reliability while providing a robust solution to the foundational problem of inter-individual signal variability due to skin tone.

Quantitative results further substantiate the impact of this approach. As shown in Table 3 and Table 4, post-gain signal amplitudes across pigmentation groups were effectively equalized, with a variance narrowed to within ±0.0008 V, compared with disparities exceeding 0.004 V prior to correction. Moreover, the regression analysis revealed a dramatic drop in the R_2_ value from 0.0816 to 0.00004, indicating that over 98% of the variance attributable to skin pigmentation was eliminated. This aligns with the visual flattening of the amplitude distribution seen in Figure 21. Importantly, this standardization not only enhances equity in signal interpretation but also bolsters diagnostic reliability. By minimizing pigmentation-induced attenuation while preserving waveform fidelity, the PGA-based system offers a robust and scalable solution for improving the inclusivity and accuracy of wearable biosensor technologies.

Additionally, the custom-designed 3D-printed enclosure provided a stable, controlled testing environment, minimizing interference from ambient light and motion artifacts. This control enabled us to isolate and analyze the true impact of skin pigmentation on signal attenuation. By pairing this environment with an automated gain control mechanism based on objective RGB skin tone measurement, we developed a novel and scalable method for improving sensor equity and reliability.

However, several limitations must be acknowledged. This study focused primarily on skin pigmentation; we did not experimentally test the system’s ability to mitigate other variables like motion artifacts or tattoos. The choice to use RGB values to infer absorption in the infrared spectrum is another limitation. While melanin, the primary determinant of skin color, is known to have broad-spectrum absorption that extends into the near-infrared range [26], the relationship is complex, and future work could benefit from directly measuring skin reflectance at the sensor’s specific wavelengths. Finally, while we collected demographic data, the sample size was insufficient for a robust analysis of covariates like age or fitness level. While this implementation showed strong performance under controlled conditions, rigorously testing the system in real-world ambulatory environments remains an important area for future work. The integration of the PGA system has been shown to significantly equalize signal amplitude across skin tones without compromising diagnostic quality, establishing a strong foundation for further development of inclusive and clinically reliable PPG sensing technologies.

## 7. Conclusions

This study successfully demonstrated a practical approach to mitigating the well-documented impact of skin pigmentation on PPG sensor signal quality. By integrating a programmable gain amplifier (PGA) controlled via microcontroller-based skin tone assessment (derived from RGB image analysis), we developed a system capable of normalizing PPG signal amplitudes across a diverse cohort of 70 human participants. The study confirmed that such dynamic gain adjustment can significantly reduce signal amplitude variability attributable to differing melanin levels, thereby promoting more equitable sensor performance.

Our findings affirm that waveform amplitude discrepancies—often a barrier to equitable sensor performance—can be effectively addressed without sacrificing the morphological features essential to clinical interpretation. Specifically, signal amplitude variance across pigmentation groups was reduced from over 0.004 V to within ±0.0008 V, while the pigmentation-to-amplitude correlation dropped from an R_2_ of 0.0816 to 0.00004, eliminating over 98% of pigmentation-induced variance. This methodology not only increases the accuracy and inclusiveness of PPG-based measurements but also opens the door for more reliable integration of optical biosensors into medical-grade wearables.

While this work establishes a strong proof of concept under controlled conditions, future research will focus on refining the skin assessment technique, exploring more granular or continuous gain control, and rigorously testing the system’s robustness in real-world ambulatory environments. Further investigation into the direct impact on SpO_2_ accuracy with gold-standard validation and the full implementation and testing on flexible substrates for true wearable applications is also a key next step. Ultimately, the proposed system lays the foundation for next-generation health monitoring tools that are both accurate and equitable across all user populations.

## Figures and Tables

**Table 1:** Component values used in the PGA circuit, corresponding to the schematic in Figure 15.

Reference	Description	Value
RF1	Feedback Resistor 1	200 kOhms
RF2	Feedback Resistor 2	2 kOhms
CF1	Feedback Capacitor 1	1.6 pF
CF2	Feedback Capacitor 2	50 pF
R1-R5	Pull-down Resistors	1 kOhms
C9, C10	LPF/Input Capacitors	100 pF
C4, C5	Decoupling Capacitors	100 nF
R11	LPF Resistor	100 Ohms

**Table 2:** Gain selection logic mapping skin tone tiers to MCU control signals and the resulting hardware state.

Gain Tier	MCU Control Signals (A1_CTRL, A2_CTRL)	Selected Feedback Path (per Figure 15)	Resulting Transimpedance Gain
Tier 1 (Highest)	(1 V—CLOSED, 0 V—OPEN)	RF1/CF1	200 k V/A
Tier 2 (Medium)	(0 V—OPEN, 1 V—CLOSED)	RF2/CF2	2 k V/A
Tier 3 (Lowest)	(0 V—OPEN, 0 V—OPEN)	None (Open)	~0 V/A

**Table 3:** Summary of signal amplitude before and after dynamic gain adjustment across skin tones.

Skin Tone	Avg. Red RGB	Mean Amp. (V) Before Gain	Mean Amp. (V) After Gain	Std. Dev. (Before)	Std. Dev. (After)
Light	235	0.0068	0.0071	0.0012	0.0009
Medium	215	0.0045	0.0069	0.0015	0.0010
Dark	185	0.0028	0.0063	0.0020	0.0011

**Table 4:** Linear regression results comparing red RGB values and signal amplitude under different test conditions.

Condition	Slope	Intercept	R^2^
Base (no gain)	0.00012	−0.0201	0.0816
Elevated (no gain)	0.00009	−0.0164	0.0716
With Gain	0.00001	0.0059	0.00004

## Data Availability

Data are contained within the article.
